# Viruses Previously Classified as CRF146_BC, a Circulating Recombinant Form of HIV-1 Recently Reported in Brazil, Represent Different Recombinant Forms, One of Which Is Circulating in Spain

**DOI:** 10.3390/v18010101

**Published:** 2026-01-12

**Authors:** Ana Donoso, María Moreno-Lorenzo, Elena Delgado, Javier E. Cañada-García, José Antonio Iribarren, Yolanda Salicio, Sonia Benito, Clara Lorente-Sorolla, Jorge Del Romero-Guerrero, María Begoña Baza-Caraciolo, Francisco Díez-Fuertes, Pilar Zamarrón, Raquel Téllez, Ana Miqueleiz, Carmen Gómez-González, Sandra Cortizo, Luis Morano, Michael M. Thomson

**Affiliations:** 1 HIV Biology and Variability Unit, Centro Nacional de Microbiología, Instituto de Salud Carlos III, 28220 Majadahonda, Madrid, Spain; ana.donoso@isciii.es (A.D.); delgade@isciii.es (E.D.);; 2 Centro de Investigación Biomédica en Red de Enfermedades Infecciosas (CIBERINFEC), Instituto de Salud Carlos III, 28220 Majadahonda, Madrid, Spain; 3 Hospital Universitario Donostia, 20014 San Sebastián, Gipuzkoa, Spain; 4 Fundación de Investigación Biomédica, Centro Sanitario Sandoval, Hospital Clínico San Carlos, 28040 Madrid, Spain; 5 Centro Sanitario Sandoval, Instituto de Medicina de Laboratorio (IML), Hospital Clínico San Carlos, 28040 Madrid, Spain; 6 AIDS Immunopathogenesis Unit, Centro Nacional de Microbiología, Instituto de Salud Carlos III, 28220 Majadahonda, Madrid, Spain; 7 Hospital Universitario Puerta de Hierro, 28222 Majadahonda, Madrid, Spain; 8 Hospital Universitario Fundación Jiménez Díaz, 28040 Madrid, Spain; 9 Hospital Universitario de Navarra, 31008 Pamplona, Navarra, Spain; 10 Hospital Universitario Araba, 01009 Vitoria, Álava, Spain; 11 Complejo Hospitalario Universitario de Vigo, 36208 Vigo, Pontevedra, Spain; 12 Hospital Universitario Álvaro Cunqueiro, 36312 Vigo, Pontevedra, Spain

**Keywords:** HIV-1, circulating recombinant forms, unique recombinant forms, genetic diversity, phylogeny, phylodynamics

## Abstract

Circulating recombinant forms (CRFs) are important components of the HIV-1 pandemic. Previous studies have reported the propagation of diverse HIV-1 CRFs of South American origin in Europe. Here, through protease-reverse transcriptase (Pr-RT) and integrase sequence analyses, we identify a Spanish cluster (BC3) branching with a Brazilian virus (10BR_RJ009) previously classified as CRF146_BC. In Pr-RT, BC3 comprised 14 viruses and was nested within a larger cluster, comprising 22 Brazilian viruses and 1 Spanish virus branching outside of BC3. Near full-length genome analyses of five BC3 viruses revealed mosaic structures identical to 10BR_RJ009, with two breakpoints delimiting a ~0.3 kb subtype B fragment within an otherwise subtype C genome. Two other Brazilian viruses previously classified as CRF146_BC (10BR_RJ039 and 01_BR_RGS69) had one and two additional short subtype B fragments, respectively, and failed to group with the 10BR_RJ009/BC3 cluster in subtype C fragments. Based on these results, we contend that 10BR_RJ009 and BC3 viruses, but not 10BR_RJ039 and 01_BR_RGS69, should be classified as CRF146_BC. Bayesian analyses estimated the CRF146_BC emergence in Brazil to be around 1999 and its introduction in Europe around 2011. CRF146_BC is the 10th CRF of South American origin reported to circulate in Europe, reflecting the relationship between South American and European HIV-1 epidemics.

## 1. Introduction

HIV-1 evolution is characterized by a high recombinogenic potential, which serves as an adaptative mechanism to confront selective pressures. Recombination in HIV-1 increases viral diversity [1], which facilitates adaptation, resulting in greater replicative fitness [2,3], and promotes immune evasion [4,5] and the evolution of drug resistance [6,7]. The high HIV-1 recombination rate has given rise to a great diversity of viral variants [8], including more than 150 circulating recombinant forms (CRFs) reported in the literature to date [9], a number that is continuously increasing through the emergence of new CRFs wherever different HIV-1 genetic forms cocirculate, and countless unique recombinant forms (URFs). The proper phylogenetic classification of HIV-1 recombinant forms through near full-length genome (NFLG) analyses is important because it allows us to better track the epidemiological spread of HIV-1 variants and because of the reported association of some CRFs with biological features [10,11,12,13] and with preferential intraclade susceptibilities to immune responses relevant to vaccines [14,15].

One of the countries where HIV-1 CRFs are generated is Brazil, where subtypes B, C, and F1 circulate, giving rise to different BF1, BC, and BCF1 recombinants. One of these is CRF146_BC, recently reported in Brazil, based on the analyses of three near full-length genomes (10BR_RJ009, 10BR_RJ039 [16], and 01_BR_RGS69 [17]) and a ~1 kb fragment in the integrase region of a fourth virus, 223_2019_BA_BR [18]. However, the authors describing this CRF noted that 01_BR_RGS69 seemed to have two short subtype B fragments in *pol* absent in the other CRF146_BC viruses, which they could detect only through informative site analyses. These fragments had been previously identified as deriving from subtype B through neighbor-joining trees by the authors first analyzing the 01_BR_RGS69 genome [17]. In spite of this, 01_BR_RGS69 was described as a virus representing CRF146_BC [18], a designation also accepted in the HIV Sequence Database, where 01_BR_RGS69 is included as one of the references of CRF146_BC [9]. Here, we (1) report the analyses of NFLG sequences of a Spanish BC recombinant cluster showing a fully coincident mosaic structure and a close phylogenetic relationship with 10BR_RJ009; (2) show with different methods (maximum likelihood, Bayesian inference, a topology test) that 01_BR_RGS69, 10BR_RJ039, and 223_2019_BA_BR viruses contain subtype B fragments absent from 10BR_RJ009; and (3) show that the subtype C fragments of 10BR_RJ039 and 01_BR_RGS69 derive, at least in part, from a parental strain different from that of 10BR_RJ009 and viruses of the Spanish BC cluster. Based on these results, we contend that only 10BR_RJ009 and the viruses of the related Spanish cluster, but not 10BR_RJ039 and 01_BR_RGS69, should be classified as genuine CRF146_BC viruses.

## 2. Materials and Methods

### 2.1. Samples

Plasma samples from HIV-1-infected individuals were collected in clinical centers from 14 Spanish regions for molecular epidemiological studies or for antiretroviral drug resistance testing.

### 2.2. PCR Amplification and Sequencing

Protease-reverse transcriptase (Pr-RT) and integrase fragments were amplified from plasma RNA by RT-PCR/nested PCR as described previously [19,20] and sequenced with the Sanger method using a capillary automated sequencer. NFLG sequences were obtained for selected samples by amplification in 5 overlapping segments from plasma RNA and sequenced by the Sanger method, as described [21,22,23]. Newly obtained sequences were deposited in GenBank, with accessions PX661829-PX661845.

### 2.3. Phylogenetic Sequence Analyses

Sequences were aligned with MAFFT v7 [24]. Initial phylogenetic trees with all Pr-RT sequences obtained by us were constructed via approximate maximum likelihood with FastTree2 [25] using the general time reversible evolutionary model with CAT approximation to account for among-site rate heterogeneity (GTR+CAT), with assessment of node support with Shimodaira-Hasegawa (SH)-like local support values [26]. Subsequently, maximum likelihood (ML) trees with sequences of interest, those branching nearest to them in the FastTree analysis, and subtype/CRF references were constructed with W-IQ-Tree [27], using the best-fit evolutionary model selected by ModelFinder [28], with assessment of node support with the ultrafast bootstrap (UFB) approximation approach [29]. Trees were viewed with MEGA v7.0 [30].

Mosaic structures were analyzed by bootscanning [31] with SimPlot v1.3.5 [32], with trees constructed using the neighbor-joining (NJ) method with the Kimura 2-parameter model and a window width of 150 or 200 nucleotides (nts) moving in 20 nt increments. Breakpoints identified with bootscanning were more precisely located at the midpoint of the transitions between clade-discriminating nts, here defined as those differing between the 75% consensuses of subtype B and of the South American subtype C strain. To further ensure the subtype affiliation of small (<200 nt) potentially recombinant segments identified with SimPlot, these were phylogenetically analyzed with relevant subtype references by maximum likelihood (ML) with W-IQ-Tree and PhyML v3 [33] and Bayesian inference with MrBayes v3.2 [34]. The analyses with PhyML were performed using the best-fit substitution model selected by Smart Model Selection [35], with assessment of node support with the approximate likelihood ratio test (aLRT), Shimodaira-Hasegawa (SH)-like procedure (aLRT-SH-like) [26], and transfer bootstrap expectation (TBE) [36]. The analyses with MrBayes were performed using the GTR+G+I substitution model, running two simultaneous independent runs and 8 chains 2–5 million generations long, ensuring that both runs reached convergence, as determined by an average standard deviation of split frequencies < 0.01. In bootscan analyses and in phylogenetic trees of short genome segments constructed to determine mosaic structures, a reconstructed BC ancestral sequence was used as the outgroup (Figure S1) to avoid artifacts caused by an outgroup too distant from the ingroup [37,38,39,40,41] derived from long-branch attraction, which may provoke distortions in the ingroup topology [37,41,42,43,44]. The confidence of the tree topologies of short recombinant segments was further assessed with the approximate unbiased (AU) test [45] using IQ-Tree [46].

### 2.4. Temporal and Geographical Estimations of Clade Ancestors

The time and location of the most recent common ancestors (MRCAs) of CRF 146_BC and the identified European cluster and its subclusters were estimated using Pr-RT sequences with the Bayesian Markov chain Monte Carlo (MCMC) coalescent method implemented in BEAST v1.10.4 [47]. Prior to performing the BEAST analysis, the temporal signal in the dataset was assessed with TempEst v.1.5.3 [48], which determines the correlation between the genetic divergence among sequences (measured as the root-to-tip distance) and time. The BEAST analysis was performed using the SRD06 codon-based evolutionary model (with two codon position partitions, 1st+2nd and 3rd) [49]. We also specified an uncorrelated lognormal relaxed clock and a Bayesian SkyGrid coalescent tree prior [50]. The MCMCs were run for 40 million generations. Mixing and convergence were checked with Tracer v1.7 [51], ensuring that the effective sample size values of all parameters were >200. We performed runs in duplicate, combining the posterior tree files with LogCombiner v1.10.4. The posterior distribution of trees was summarized in a maximum clade credibility (MCC) tree with TreeAnnotator v1.10.4, after removal of a 10% burn-in. MCC trees were visualized with FigTree v1.4.2 (Rambaut, http://tree.bio.ed.ac.uk/software/figtree/, accessed on 1 January 2026). Parameter uncertainty was summarized in 95% highest posterior density (95% HPD) intervals.

## 3. Results

### 3.1. Phylogenetic Analyses of Pr-RT and Integrase

**Figure 1:**
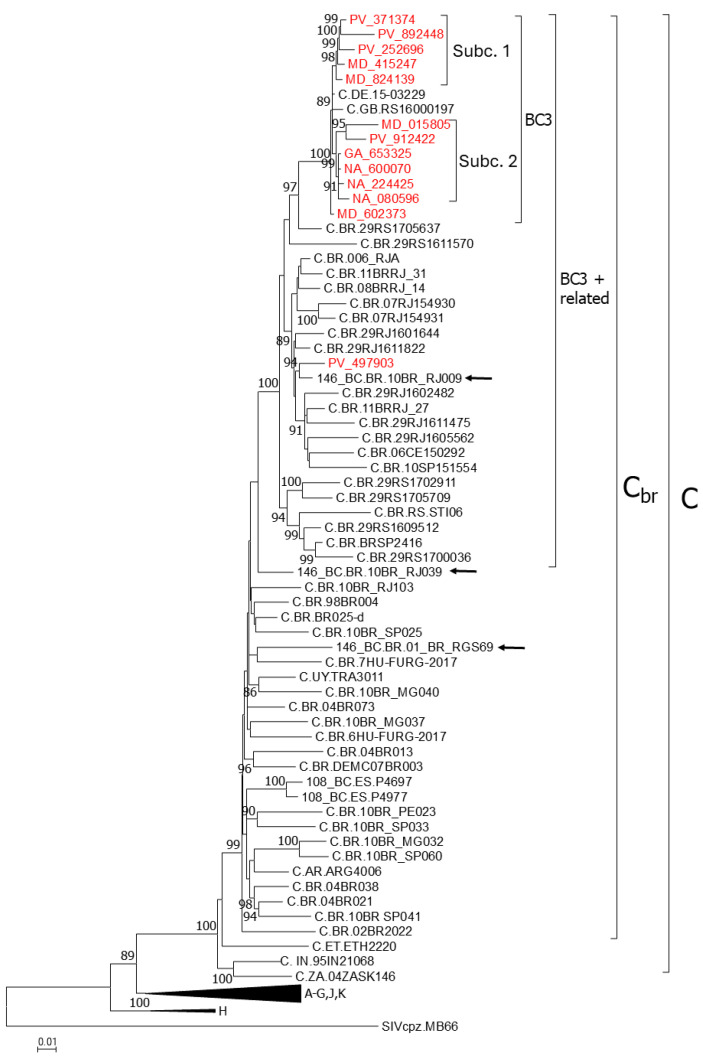
ML tree of Pr-RT showing phylogenetic relationships of the BC3 cluster. In addition to BC3 viruses, the tree includes references of all HIV-1 group M subtypes, all subtype C viruses from South America sequenced in NFLG available at the HIV Sequence Database, references of CRF_BCs of Brazilian ancestry which are nonrecombinant subtype C in Pr-RT (CRF108_BC and CRF146_BC), and sequences that in a previous analysis with FastTree grouped with BC3. Names of viruses from Spain sequenced by us are in red. Database subtype C and BC recombinant viruses are named with their genetic form, as classified in the HIV Sequence Database, followed by the country of sample collection’s 2-letter ISO code, and the name of the virus. Viruses classified as CRF146_BC in the HIV Sequence Database are indicated with arrows. Clades corresponding to the BC3 cluster, BC3+related viruses, the Brazilian subtype C strain, and subtype C are indicated with brackets. Clades corresponding to HIV-1 group M non-subtype C references are compressed. Node support values represent UFB values. Only values ≥ 80% are shown.

Through phylogenetic analyses of Pr-RT sequences from HIV-1-infected individuals attending Spanish clinical centers, we identified a subtype C cluster related to the Brazilian subtype C strain, comprising sequences from 12 individuals from four Spanish regions. Since in the sequenced integrase genes these viruses were BC recombinant (see below), the cluster was designated BC3. All individuals with viruses in the BC3 cluster were men, infected by sexual contact, predominantly men who have sex with men (MSM), predominantly native Spaniards, and diagnosed with HIV-1 infection in 2017–2024 (Table 1). Through searches with the basic local alignment search tool (BLAST 2.17.0) at the Los Alamos HIV Sequence Database and subsequent phylogenetic analyses, we found two additional Pr-RT sequences, from the United Kingdom (UK) and Germany, respectively, branching within the BC3 cluster. This cluster comprised two Spanish subclusters (subcluster 1, *n* = 5; subcluster 2, *n* = 6), with the viruses from UK and Germany branching outside of them (Figure 1). All five individuals harboring viruses from subcluster 1 were MSM, while three of six infections in subcluster 2 were heterosexually acquired. Through phylogenetic analyses including all sequences from viruses classified at the Los Alamos database as being of subtype C or BC recombinant from South America and Europe, we found that the Spanish cluster was nested within a larger cluster comprising 22 Brazilian viruses, including 10BR_RJ009, a virus previously classified as CRF146_BF1, and one virus from Spain sequenced by us, PV_497903, from a Spanish individual, branching interspersed among the Brazilian viruses (Figure 1). Of the two other viruses previously classified as CRF146_BC, 10BR_RJ039 branched basally to the clade comprising BC3 and related Brazilian viruses, joining it with a relatively low UFB support (78%), and 01_BR_RGS69 branched interspersed among Brazilian subtype C viruses (Figure 1).

**Figure 2:**
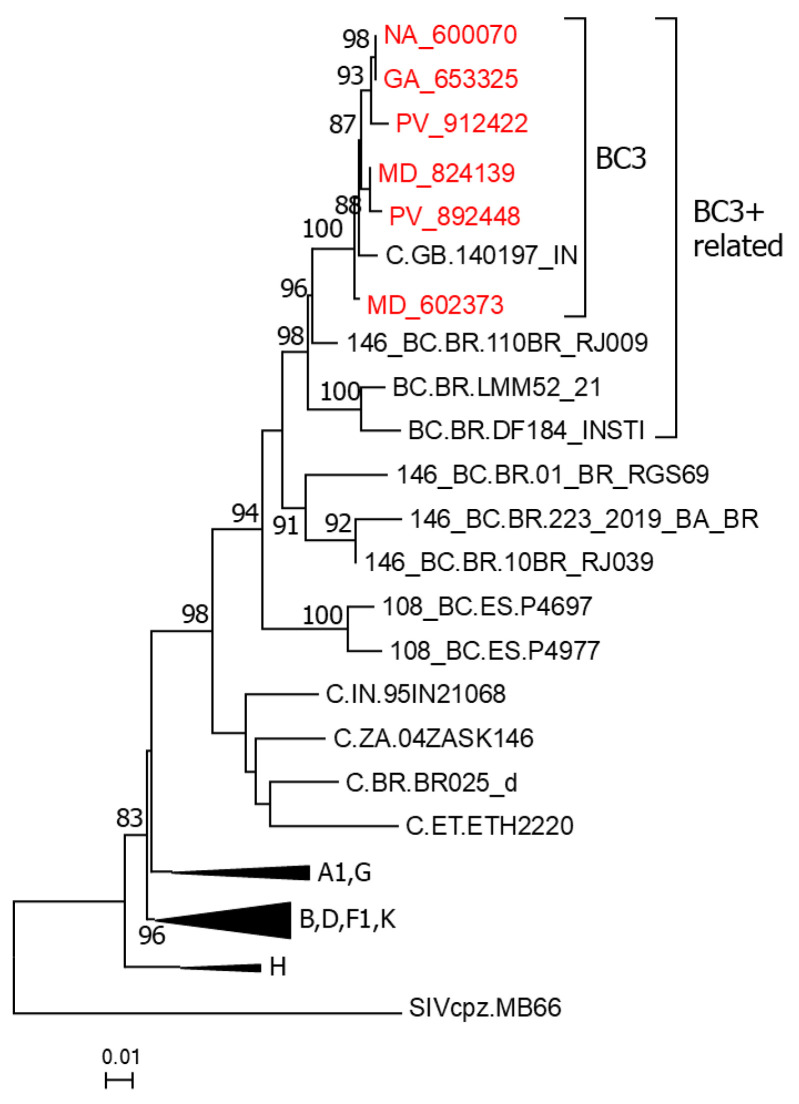
ML tree of integrase showing phylogenetic relationships of the BC3 cluster. In addition to BC3 viruses, the tree includes references of all HIV-1 group M subtypes, references of CRF_BCs of Brazilian ancestry which are BC recombinant in integrase (CRF108_BC and CRF146_BC), and sequences that in a previous analysis with FastTree grouped with BC3. Names of viruses from Spain sequenced by us are in red. Database subtype C and BC recombinant viruses are named with their genetic form, as classified in the HIV Sequence Database, followed by the country of sample collection’s 2-letter ISO code, and the name of the virus. Clades corresponding to the BC3 cluster and BC3+related viruses are indicated with brackets. Clades corresponding to HIV-1 group M non-subtype C references are compressed. Node support values represent UFB values. Only values ≥ 80% are shown.

From six of the viruses sequenced by us in Pr-RT, we obtained sequences of integrase, where they also grouped, together with one virus from the UK (which is the same grouping in BC3 in Pr-RT), in a phylogenetic cluster, nested within a larger cluster comprising 10BR_RJ009 and two other Brazilian BC recombinant viruses (Figure 2). In the integrase tree, 10BR_RJ039, 01_BR_RGS69, and 223_2019_BA_BR grouped in a clade which branched as a sister clade to the clade of BC3+related Brazilian viruses, but the UFB value supporting the relationship of both clades was low (59%). In bootscan analyses, the integrase sequences of the Spanish cluster and the related Brazilian viruses exhibited a BC recombinant structure, with one breakpoint at a coincident position; 10BR_RJ039, 01_BR_RGS69, and 223_2019_BA_BR had breakpoints at the same position but appeared to have a second breakpoint in the 5′ segment of integrase (Figure S2).

### 3.2. Phylogenetic Analyses of NFLG Sequences and of Separate Subtype C and B Fragments

**Figure 3:**
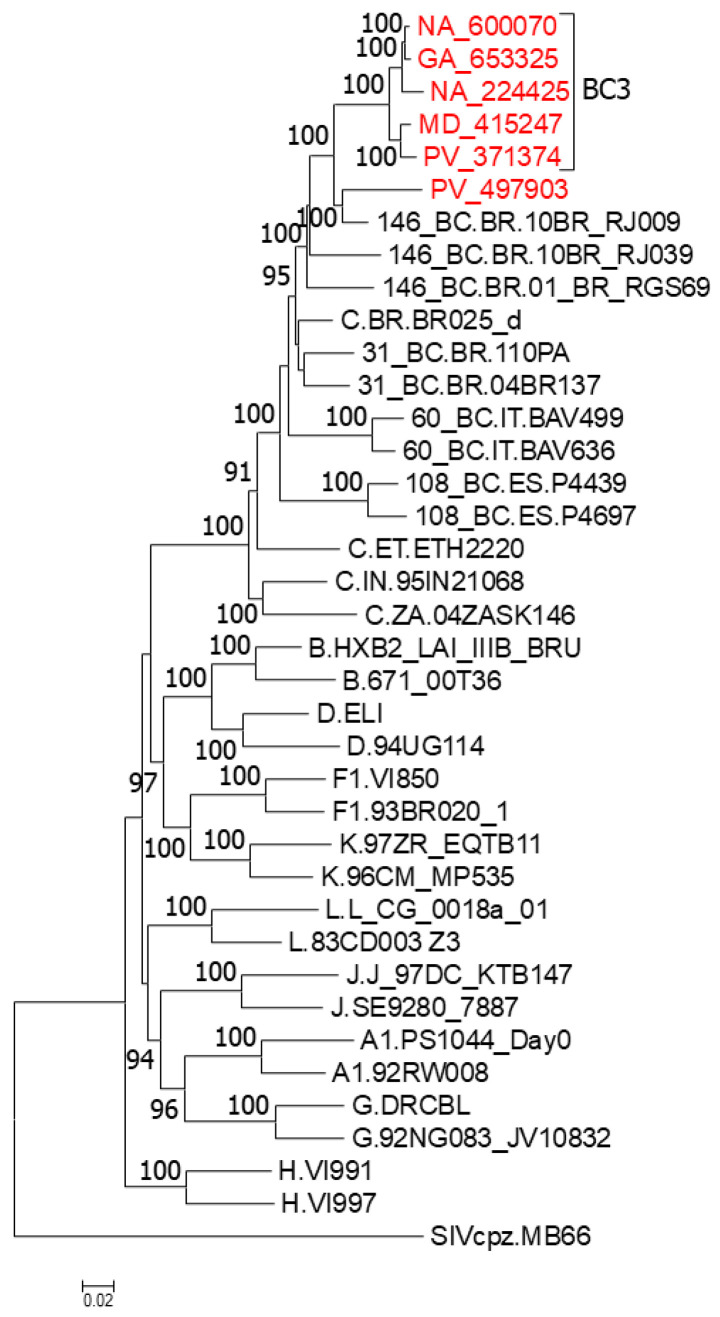
ML tree of NFLG showing phylogenetic relationships of the BC3 cluster. In addition to BC3 viruses, the tree includes references of all HIV-1 group M subtypes and of all CRF_BCs of Brazilian ancestry (CRF31_BC, CRF60_BC, CRF108_BC, and CRF146_BC). Names of viruses from Spain sequenced by us are in red. Database subtype C and CRF_BC viruses are named with the genetic form, as classified in the HIV Sequence Database, followed by the country of sample collection’s 2-letter ISO code, and the name of the virus. Node support values represent UFB values. Only values ≥ 90% are shown.

We obtained NFLG sequences of five viruses from the BC3 cluster and of the related PV_497903 virus. In the phylogenetic tree, all six viruses grouped in a clade, together with the Brazilian viruses 10BR_RJ009, 10BR_RJ039, and 01_BR_RGS69, previously classified as CRF146_BC, with PV_497903 and 10BR_RJ009 grouping together in a sister clade of BC3, and 10BR_RJ039 and 01_BR_RGS69 branching basally to the other viruses (Figure 3).

**Figure 4:**
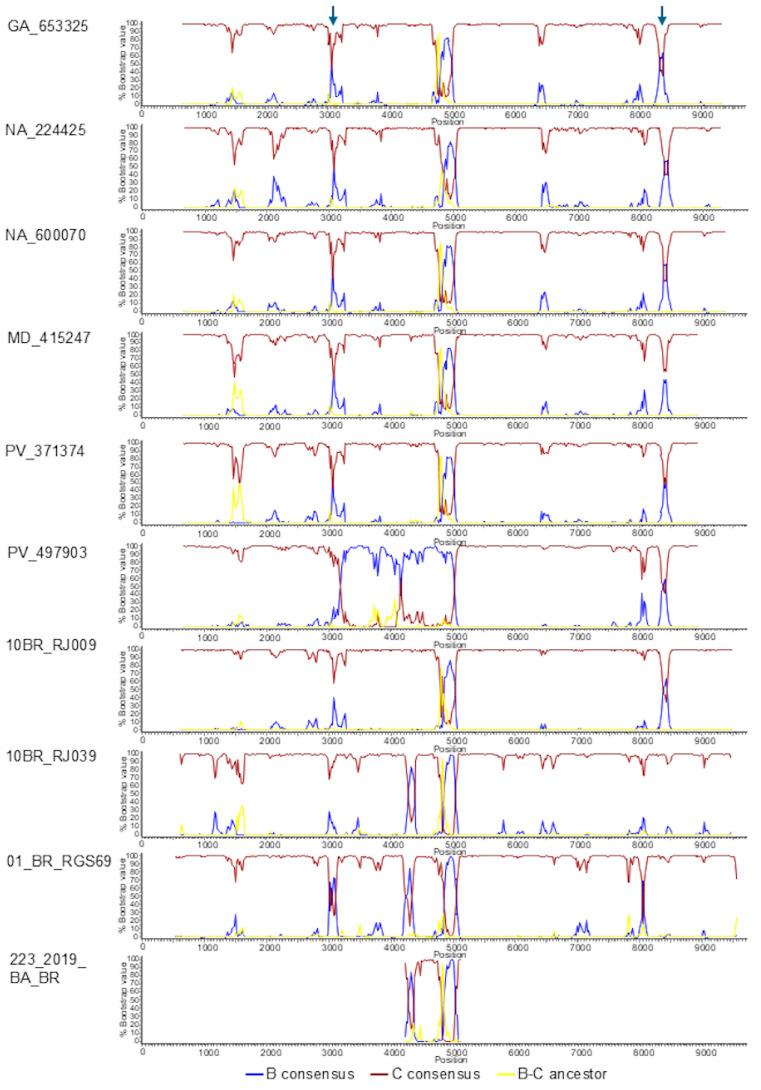
Bootscan analyses of NFLG of BC3 cluster viruses, of viruses previously classified as CRF146_BC, of a virus from Spain (PV_497903) closely related to BC3, and of the available ∼1 kb sequence of the CRF146_BC-classified virus 223_2019_BA_BR. The horizontal axis represents HXB2 nt positions, and the vertical axis represents the bootstrap values with which the query sequence groups with the reference sequences, which are 75% consensuses of subtype B and the Brazilian subtype C strain, and a reconstructed BC ancestor used as an outgroup. Trees were constructed with the neighbor-joining method using Kimura 2-parameter distances. A window of 200 nt was used, except for 10BR_RJ039, 01_BR_RGS069, and 223_2019_BA_BR, for which a window of 150 nt was used, moving in 20 nt increments. The vertical arrows on top point to two segments where support for grouping with subtype B increased in BC3 and the related viruses 10BR_RJ009 and PV_497903, but whose subtype B affiliation was not supported in ML trees.

**Figure 5:**
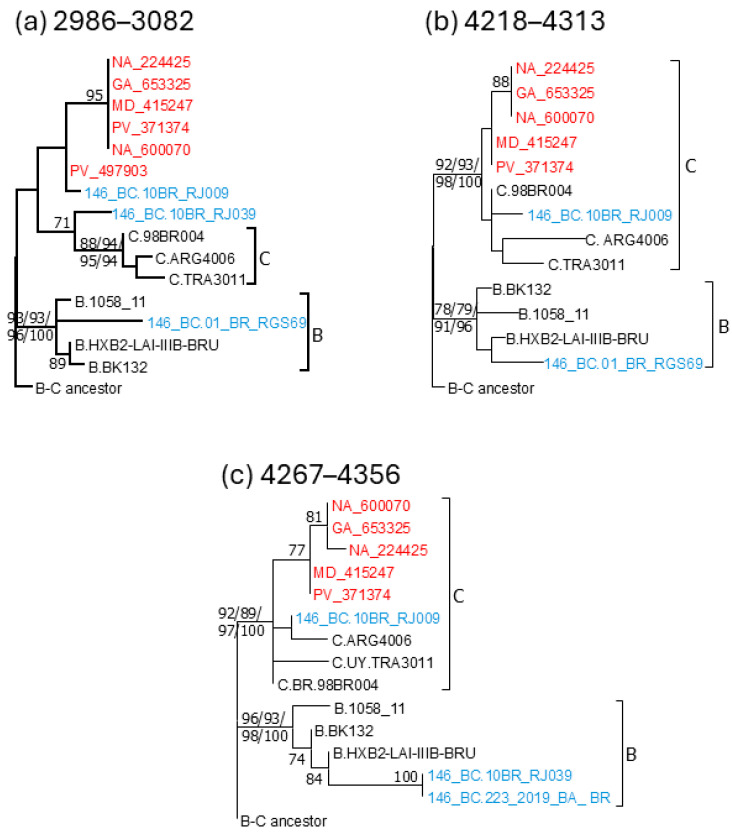
Phylogenetic trees of 3 genome segments where bootscan analyses suggested a subtype B affiliation in 01_BR_RGS69 (2986–3082, 4218–4313) and 10BR_RJ0039 and 223_2019_BA_BR (4267–4356) not observed in BC3 or 10BR_RJ009 viruses. HXB2 positions delimiting the analyzed segments are shown above each tree. Names of viruses from Spain sequenced by us are in red, and viruses previously classified as CRF146_BC are in blue. Trees were rooted with a reconstructed BC ancestor sequence. Node support values corresponding to the B and C clades were obtained with 4 different methods and are shown in this order—UFB/aLRT SH-like/TBE/Bayesian PP—all expressed as percentages. In other nodes, only UFB values are shown. Only support values ≥ 70% are shown.

Bootscan analyses of the five sequenced BC3 genomes showed a recombinant structure with a largely subtype C genome and two breakpoints in integrase, at HXB2 positions 4812 and 5076 (as located in the midpoint of the transitions of subtype-discriminating nts), delimiting a subtype B fragment, a structure that was coincident with that of 10BR_RJ009 (Figure 4). PV_497903 exhibited a different mosaic structure with a larger subtype B fragment delimited by breakpoints at 3199 and 5076 HXB2 positions (Figure 4). The subtype B segment at the 4812–5076 positions found in BC3 and 10BR_RJ009 viruses was also observed in the bootscan analyses of 10BR_RJ039 and 01_BR_RGS69, which also suggested the presence of additional short subtype B segments in these viruses: one in integrase in 10BR_RJ039 and two in integrase and RT, respectively, in 01_BR_RGS69. Phylogenetic trees using four different methods (ML with assessment of node support with UFB, aLRT-SH-like, and TBE methods, and Bayesian inference) confirmed the subtype B affiliation of the 4267–4356 (integrase) segment in 10BR_RJ039 and of the 2986–3082 (RT) and 4218–4313 (RT-integrase junction) segments in 01_BR_RGS69 (with positions numbered according to the HXB2 genome) (Figure 5). The bootscan analysis of 233_2019_BA_BR virus, for which only the sequences of a ~1 kb fragment comprising integrase and adjacent segments are available, showed a recombinant structure coincident with 10BR_RJ039 (Figure 4), with the 4267–4356 subtype B fragment also supported in phylogenetic trees (Figure 5). Further support for the subtype B affiliation of the short subtype B segments of 10BR_RJ039, 01_BR_RGS69, and 223_2019_BA_BR detected by bootscanning and phylogenetic analyses was obtained through AU tree topology tests, which rejected the topologies in which these segments clustered with subtype C (*p* = 0.001 in the 4267–4356 segment of 10BR_RJ039 and 23_2019_BA_BR; *p* = 0.036 and *p* = 0.025, respectively, in the 2986–3082 and 4218–4313 segments of 01_BR_RGS69).

**Figure 6:**
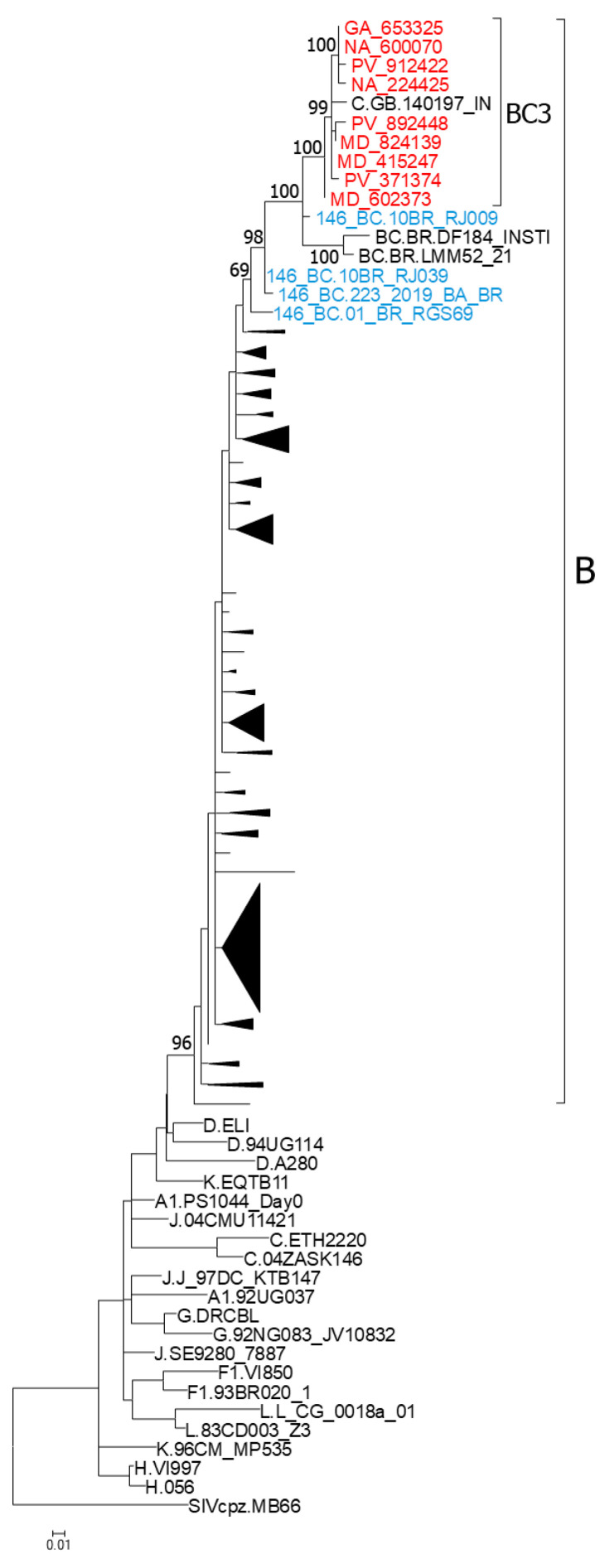
ML tree of the 4812–5075 genome fragment of viruses of the BC3 cluster, viruses previously classified as CRF146_BC, and 3 database viruses grouping with BC3 in integrase. The tree includes references of all HIV-1 group M subtypes and all subtype B viruses from Brazil sequenced in NFLGs available in the HIV Sequence Database. Names of viruses from Spain sequenced by us are in red, and those previously classified as CRF146_BC are shown in blue. Node support values represent UFB values. Only values of the nodes corresponding to clades comprising BC3 and viruses classified as CRF146_BC are shown. Names of viruses from Spain sequenced by us are in red. Subclades of subtype B viruses are shown compressed as triangles.

**Figure 7:**
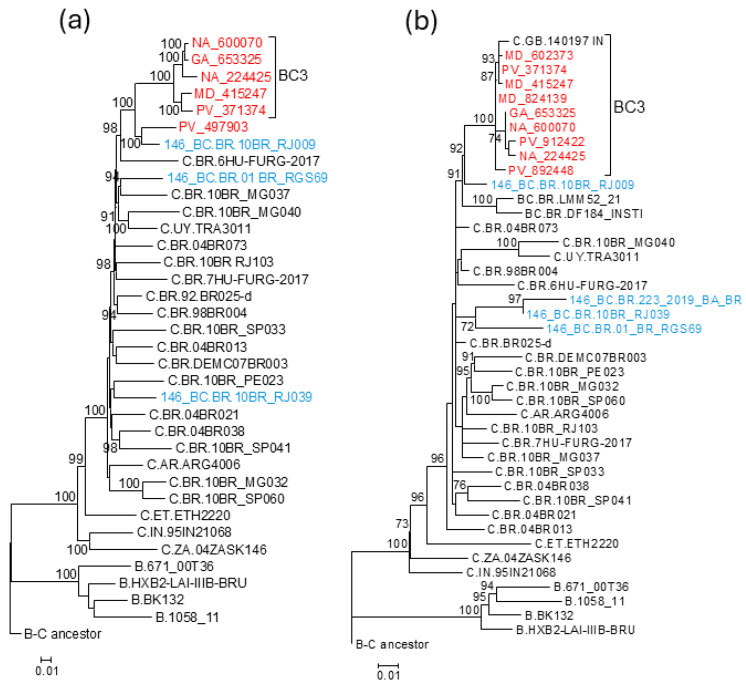
ML tree of (**a**) concatenated subtype C NFLG fragments of BC3 viruses, the related PV_497903 virus, and viruses previously classified as CRF146_BC, and (**b**) concatenated subtype C fragments in integrase of BC3 viruses, viruses previously classified as CRF146_BC, and two other viruses from Brazil grouping with BC3 in integrase. The tree includes references of subtypes B and C and sequences of all subtype C viruses from South America sequenced in NFLG available at the HIV Sequence Database. Names of viruses from Spain sequenced by us are in red, and those previously classified as CRF146_BC are in blue. Node support values represent UFB values. Only values ≥ 70% are shown. The trees are rooted with a reconstructed ancestral BC sequence.

To examine whether the parental strains of the analyzed BC recombinants had a common ancestry, we constructed separated phylogenetic trees of the 4812–5076 subtype B segment and of the concatenated subtype C genome fragments shared by all BC recombinant viruses here analyzed, together with South American subtype B and C viruses used as controls. In the subtype B fragment, viruses from the BC3 cluster, 10BR_RJ009, 10BR_RJ039, and three Brazilian viruses sequenced in integrase (223_2019_BA_BR, LMM52_21, and DF184_INSTI) grouped in a clade supported by a 98% UFB value, while 01_BR_RGS69 branched as an outlier of this clade, joining it with a relatively low UFB support (69%) (Figure 6). In the tree of concatenated subtype C fragments, 10BR_RJ009 and PV_497903 branched with the BC3 cluster with a 100% UFB support. However, 10BR_RJ039 and 01_BR_RGS69 were unrelated to this cluster, branching interspersed among Brazilian subtype C viruses (Figure 7a). Similarly, in a tree of concatenated subtype C fragments of integrase, BC3 viruses, 10BR_RJ009, and two other BC recombinant viruses from Brazil (LMM52_21 and DF184_INSTI) branched in a clade that did not include 01_BR_RGS69, 10BR_RJ039, and 223_2019_BA_BR, which branched interspersed among Brazilian subtype C viruses (Figure 7b).

In the trees of integrase and concatenated subtype C fragments of integrase, a close relationship between 10BR_RJ039 and 223_2019_BA_BR was observed (Figure 2 and Figure 7b).

**Figure 8:**
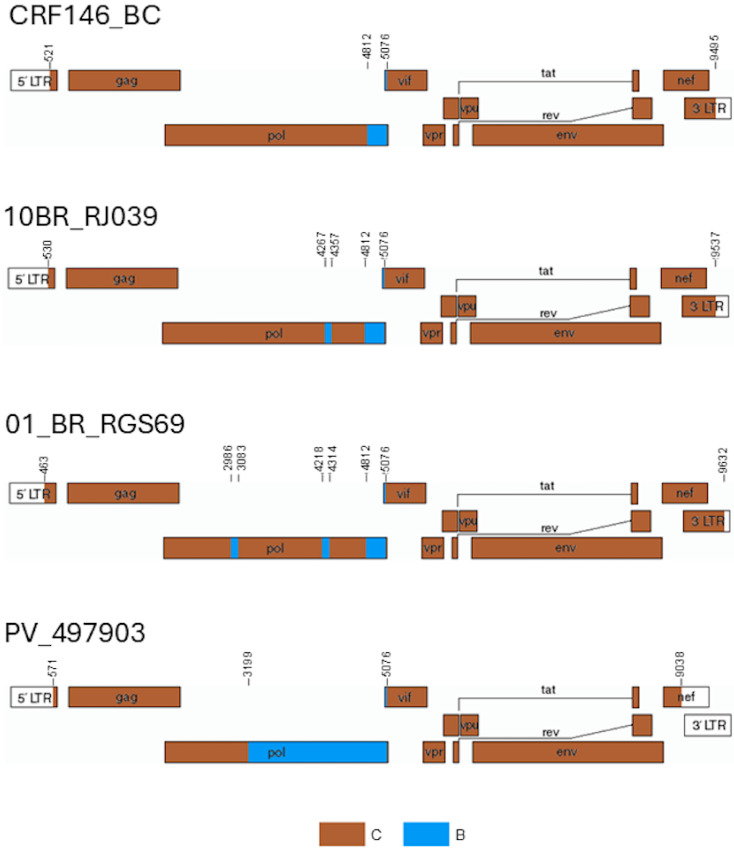
Depictions of the mosaic structures of CRF146_BC viruses, as designated in this study (viruses of the BC3 cluster and 10BR_RJ009), of the viruses previously classified as CRF146_BC 10BR_RJ039 and 01_BR_RGS69, and of the unique recombinant from (URF) 146B virus PV_497903.

These analyses, therefore, indicate a close relationship and coincidence of mosaic structures of BC3 viruses and 10BR_RJ009. However, 10BR_RJ039 and 01_BR_RGS069 contain additional subtype B fragments and seem to derive from different parental subtype C strains. Therefore, we propose that, among the BC recombinants sequenced in NFLG here analyzed, only 10BR_RJ009 and BC3 viruses should be classified as CRF146_BC. Depictions of the mosaic structures of CRF146_BC and the other BC recombinants here analyzed are shown in Figure 8.

### 3.3. Phylodynamic Analysis

**Figure 9:**
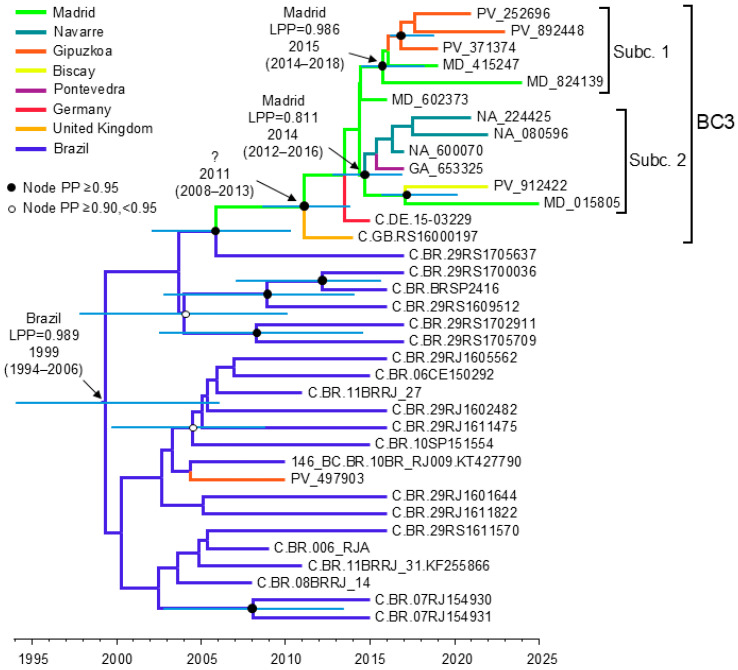
Maximum clade credibility tree of Pr-RT sequences of BC3 and related viruses, presumably representing CRF146_BC. Branch colors indicate, for terminal branches, country of sample collection, and for internal branches, the most probable location country of the subtending node, according to the legend on the upper left. Nodes supported by PP ≥ 0.95 are marked with filled circles, and those with PP ≥ 0.90 and <0.95 with unfilled circles. The most probable locations at the root of the tree and at the nodes corresponding to the BC3 cluster and its 2 subclusters are indicated, together with the PPs supporting each location (LPPs) and the time of the MRCA (mean value, with 95% HPD interval in parentheses). The 95% HPD intervals of the times of MRCA corresponding to nodes supported by PP ≥ 0.90 are indicated with horizontal bars. The ? symbol at the node of the BC3 cluster indicates uncertainty in the location reflected in low (<0.5) LPP support.

The TempEst analysis indicated the existence of an adequate temporal signal in the Pr-RT dataset (r^2^ = 0.4396, which increased to 0.6547 after an outlier sequence from Brazil was removed). The Bayesian coalescent analysis estimated a substitution rate of 2.59 × 10^−3^ subs./site/year. The emergence of the cluster presumably representing CRF146_BC (including BC3, 10BR_RJ009, and related viruses) was estimated in Brazil around 1999, and its introduction in Europe (represented by the BC3 cluster) was estimated around 2011 (Figure 9). However, the place of origin of BC3 could not be estimated with confidence, since the highest location PP was only 0.409. The analysis supported the origin of both BC3 subclusters in Madrid, around 2014 and 2015, respectively.

## 4. Discussion

The results of this study show that the four Brazilian viruses previously classified as HIV-1 CRF146_BC (10BR_RJ009, 10BR_RJ039, 01_BR_RGS69, and 223_2019_BA_BR) [9,18] represent three different recombinant forms, one of which (represented by 10BR_RJ009) belongs to the same recombinant form as a BC recombinant cluster newly identified in Spain (designated BC3) of which we obtained five NFLG sequences. The simplest of the analyzed mosaic structures corresponds to 10BR_RJ009 and the BC3 cluster viruses, having only two breakpoints delimiting a subtype B segment in integrase (HXB2 positions 4812–5076), with the rest of the genome deriving from subtype C. The identity of the recombinant forms of 10BR_RJ009 and the BC3 cluster is further reinforced by clustering in separate phylogenetic trees of subtype B and subtype C segments (Figure 6 and Figure 7). 10BR_RJ039, sequenced in NFLG, and 223_2019_BA_BR, sequenced in only ~1 kb in the integrase region, have, in addition to the 4812–5076 subtype B segment, a second subtype B segment in integrase (4267–4356 HXB2 positions) (Figure 4 and Figure 5). The close phylogenetic relationship of 10BR_RJ039 and 223_2019_BA_BR is also supported in a tree of concatenated subtype C fragments in integrase (Figure 7b). 01_BR_RGS69 has, in addition to the 4812–5076 subtype B segment, two other subtype B segments in RT (2986–3082 HXB2 positions) and across the RT-integrase junction (4218–4313 HXB2 positions) (Figure 6 and Figure 7), whose existence was previously suggested through NJ phylogenetic and informative site analyses, and is here confirmed through ML and Bayesian phylogenetic analyses and a tree topology test. Besides the differences in mosaic structures, the distinctiveness of the recombinant forms represented by the 10BR_RJ009+BC3 cluster, 10BR_RJ039+223_2019_BA_BR, and 01_BR_RGS69 is also supported by separate branching in trees of concatenated subtype C fragments (Figure 6 and Figure 7).

Based on these results, we contend that, among the viruses here analyzed, only 10BR_RJ009 and BC3 viruses represent a circulating recombinant form, which, according to the previous designation of 10BR_RJ009, should be designated CRF146_BC. 10BR_RJ039 and 223_2019_BA_BR, given their close phylogenetic relation and coincident recombinant structures, could represent another potential CRF, but its definition would require obtaining at least one additional NFLG of a virus sharing the same mosaic structure and parental strains. 01_BR_RGS69, with the data currently available, should be classified as a BC URF.

We also obtained the NFLG sequence of a sixth BC recombinant virus from Spain, PV_497903, branching in Pr-RT basally to the BC3 cluster, interspersed among Brazilian viruses. PV_497903′s mosaic structure differed from that of BC3 and 10BR_RJ009 viruses, with a longer subtype B segment in *pol*, between HXB2 positions 3199 and 5076. Sincein concatenated subtype C fragments PV_497903 groups with 10BR_RJ009, we posit that the virus derives from secondary recombination of a Brazilian CRF146_BC virus with a subtype B virus.

In the study originally describing CRF146_BC [18], the authors reported that 10BR_RJ009, 10BR_RJ039, 01_BR_RGS69, and 223_2019_BA_BR grouped in a well-supported clade in concatenated subtype C segments in integrase and contiguous segments of RT and *vif* (4172–4834 + 5071–5190 HXB2 positions) and in the subtype B fragment of integrase (4833–5066 HXB2 positions), assessing node supports with aLRT and bootstrapping. However, we could not confirm these results, as the RT-INT-vif concatenated sequences of 10BR_RJ039, 01_BR_RGS69, and 223_2019_BA_BR failed to group with those of the clade formed by 10BR_RJ009 and the BC3 cluster (Figure S3), and in the integrase subtype B fragment, a phylogenetic relationship with the 10BR_RJ009+BC3 cluster was only supported for 10BR_RJ039 and 223_2019_BA_BR, but not for 01_BR_RGS69 (Figure S4). We will not speculate on the causes of the discrepant results, although we note that we included as controls all NFLG sequences of the South American subtype C strain and of Brazilian subtype B sequences available at the HIV Sequence Database, which coincide only partially with those used by Oliveira et al. [18]. We also note that these authors did not perform a phylogenetic analysis of all concatenated subtype C sequences common to the CRF146-like recombinant NFLGs including multiple control sequences from the South American subtype C strain.

In analyses of Pr-RT (Figure 1) and integrase (Figure 2), we identify 24 additional Brazilian viruses branching in the CRF146_BC clade that, therefore, could belong to this CRF, which indicates that CRF146_BC is more common in Brazil than previously reported, although sequencing of NFLG would be needed for definitive confirmation. We also identify two UK viruses and one German virus branching in the CRF146_BC clade, more specifically in the BC3 cluster, although they could not be linked to Spain, since they branched outside of the Spanish subclusters, and Bayesian coalescent analyses failed to confidently estimate a geographic origin of the BC3 cluster (Figure 9).

Short (<100 nt) recombinant segments, as found in 10BR_RJ039, 223_2019_BA_BR, and 01_BR_RGS69, have been reported in multiple HIV-1 CRFs, even as short as 42 nt in CRF63_02A6 [52] or 54 nt in three closely related Cuban BG CRFs 20, 23, and 24_BG [53], identified through ML phylogenetic analyses and, in the case of the Cuban CRFs, also by tree topology testing and the detection of a unique combination of three rare nts exclusive of these CRFs and their parental subtype B strain. A precise genetic characterization of HIV-1 recombinant forms may be important, since even relatively minor differences may result in significant biological differences, as exemplified by frequent CXCR4 coreceptor usage in CRF14_BG, associated with only four amino acid residues in the Env V3 loop [10], absent in the closely related CRF73_BG [54], and the presence in CRF122_BF1, but not in the closely related CRF72_BF1, of an amino acid residue in protease reported to contribute to drug resistance [55].

CRF146_BC is the 10th CRF of South American ancestry identified in samples collected in Europe through NFLG analyses, after CRF12_BF [22], CRF42_BF1 [56], CRF47_BF1 [57], CRF60_BC [58], CRF66_BF [59], CRF75_BF1 [13], CRF89_BF1 [60], CRF108_BC [23], and CRF122_BF1 [55], which reflects the relationship between the South American and European HIV-1 epidemics, probably derived from migratory fluxes between both continents.

It is interesting to note that the Spanish CRF146_BC cluster (BC3) comprised two subclusters that appeared to be associated with different transmission routes: all five individuals from subcluster 1 were MSM and at least three of six infections from subcluster 2 were heterosexually acquired. Since the earliest HIV-1 diagnoses (one in 2016 and two in 2017) are from subcluster 2, corresponding to two heterosexually acquired infections and one infection with unspecified sexual transmission, it could be speculated that CRF146_BC would have spread in Spain from a heterosexual-driven to an MSM-driven network, which, considering that sex among men is the main transmission mode of HIV-1 in Spain, could facilitate its spread in the country. A similar observation was reported in Spain for three other CRFs of South American origin, CRF47_BF1 [40], CRF66_BF1 [59], and CRF89_BF1 [60]. Such a phenomenon may reflect the migration of these CRFs from countries with predominantly heterosexual transmission to Spain, where most HIV-1 clusters are associated with MSM [61,62]. It should be pointed out, however, that all 12 individuals from Spain from the BC3 cluster were male, which casts doubt on the self-reported heterosexual HIV-1 acquisitions, raising the possibility that at least some of them could be nondisclosed MSM [63,64].

Frequently, new CRFs have been originally identified as clusters branching outside of known CRF clades, usually detected through analyses of Pr-RT sequences obtained for drug resistance testing. More recently, the analyses of integrase sequences, obtained to detect resistance to integrase inhibitors, have allowed the identification of recombinants that would go unnoticed through Pr-RT analyses, as are the cases of CRF146_BC or CRF108_BC [23]. This suggests that the proportions of CRFs or URFs estimated in molecular epidemiological surveys [65], mostly based on Pr-RT sequences, represent gross underestimates, which underscores the need for the NFLG sequencing of representative samples of new HIV-1 diagnoses for more accurate estimations of the real prevalence of recombinant forms.

## 5. Conclusions

The results presented here show that four Brazilian HIV-1 viruses previously classified as CRF146_BC represent three different recombinant forms, one of which, represented by 10BR_RJ009, is circulating in Spain, with sporadic cases in the UK and Germany phylogenetically related to the Spanish viruses. Based on these results, we contend that, among viruses analyzed in NFLG, only 10BR_RJ009 and viruses of the related European cluster with coincident mosaic structures should be classified as CRF146_BC. The results also indicate that CRF146_BC is more common in Brazil than previously reported, as suggested by the branching of 24 Brazilian viruses with CRF146_BC in Pr-RT or integrase.

## Figures and Tables

**Table 1:** Epidemiological data of patients studied by us.

Sample ID	City of Sample Collection	Region of Sample Collection	Year of Sample Collection	Year of HIV Diagnosis	Gender	Transmission Route *	Country/ Geographical Area of Origin
GA_653325	Vigo	Galicia	2017	2017	M	HT	Spain
MD_015805	Madrid	Madrid	2024	2024	M	MSM	Spain
MD_415247	Madrid	Madrid	2019	2018	M	MSM	Spain
MD_602373	Madrid	Madrid	2016	2015	M	Sexual	Spain
MD_824139	Madrid	Madrid	2024	2022	M	MSM	South America
NA_080596	Pamplona	Navarre	2022	2022	M	HT	Spain
NA_224425	Pamplona	Navarre	2021	2021	M	HT	Spain
NA_600070	Pamplona	Navarre	2017	2017	M	HT	South America
PV_252696	San Sebastián	Basque Country	2021	2021	M	MSM	Central America
PV_371374	San Sebastián	Basque Country	2019	2019	M	MSM	Spain
PV_497903	San Sebastián	Basque Country	2010	2010	M	MSM	Spain
PV_892448	San Sebastián	Basque Country	2023	2023	M	MSM	Spain
PV_912422	Vitoria	Basque Country	2022	2022	M	Sexual	Spain

* HT: heterosexual; MSM: men who have sex with men; Sexual: unspecified sexual.

## Data Availability

The sequences newly obtained in the study are available in GenBank under accessions PX661829-PX661845.

## Supplementary Materials

The following supporting information can be downloaded at: https://www.mdpi.com/article/10.3390/v18010101/s1, Figure S1: Phylogenetic tree of NFLG sequences of HIV-1 group M subtype references used to reconstruct the B-C ancestral sequence; Figure S2: Bootscan analyses of integrase sequences of BC3 cluster and related viruses, and of viruses classified as CRF146_BC at the HIV Sequence Database; Figure S3: Phylogenetic trees of concatenated subtype C fragments in integrase and surrounding segments of BC3 cluster viruses and viruses previously classified as CRF146_BC; Figure S4: Phylogenetic trees of the subtype B fragment of integrase of BC3 cluster and related viruses and viruses previously classified as CRF146_BC.

## Abbreviations

The following abbreviations are used in this manuscript:

aLRT SH-like	Approximate likelihood ratio test, Shimodaira-Hasegawa-like procedure
AU	Approximate unbiased
CRF	Circulating recombinant form
GTR+CAT	General time reversible with CAT model
GTR+G+I	General time reversible with gamma-distributed rate variation among sites and a proportion of invariable sites
HIV-1	Human immunodeficiency virus type 1
HPD	Highest posterior density
MCC	Maximum clade credibility
ML	Maximum likelihood
MRCA	Most recent common ancestor
MSM	Men who have sex with men
NFLG	Near full-length genome
PP	Posterior probability
Pr-RT	Protease-reverse transcriptase
RT	Reverse transcriptase
RT-PCR	Reverse transcription-polymerase chain reaction
TBE	Transfer bootstrap expectation
UFB	Ultrafast bootstrap
URF	Unique recombinant form
