# Supplementary material for: Viruses Previously Classified as CRF146_BC, a Circulating Recombinant Form of HIV-1 Recently Reported in Brazil, Represent Different Recombinant Forms, One of Which Is Circulating in Spain
Source: Viruses. 2026 Jan 12;18(1):101. doi: 10.3390/v18010101 (PMC12846359; doi:10.3390/v18010101)
Supplement: Supplementary file 1 [file viruses-18-00101-s001.zip › viruses-4059699-supplementary.pdf]

Supplementary figures

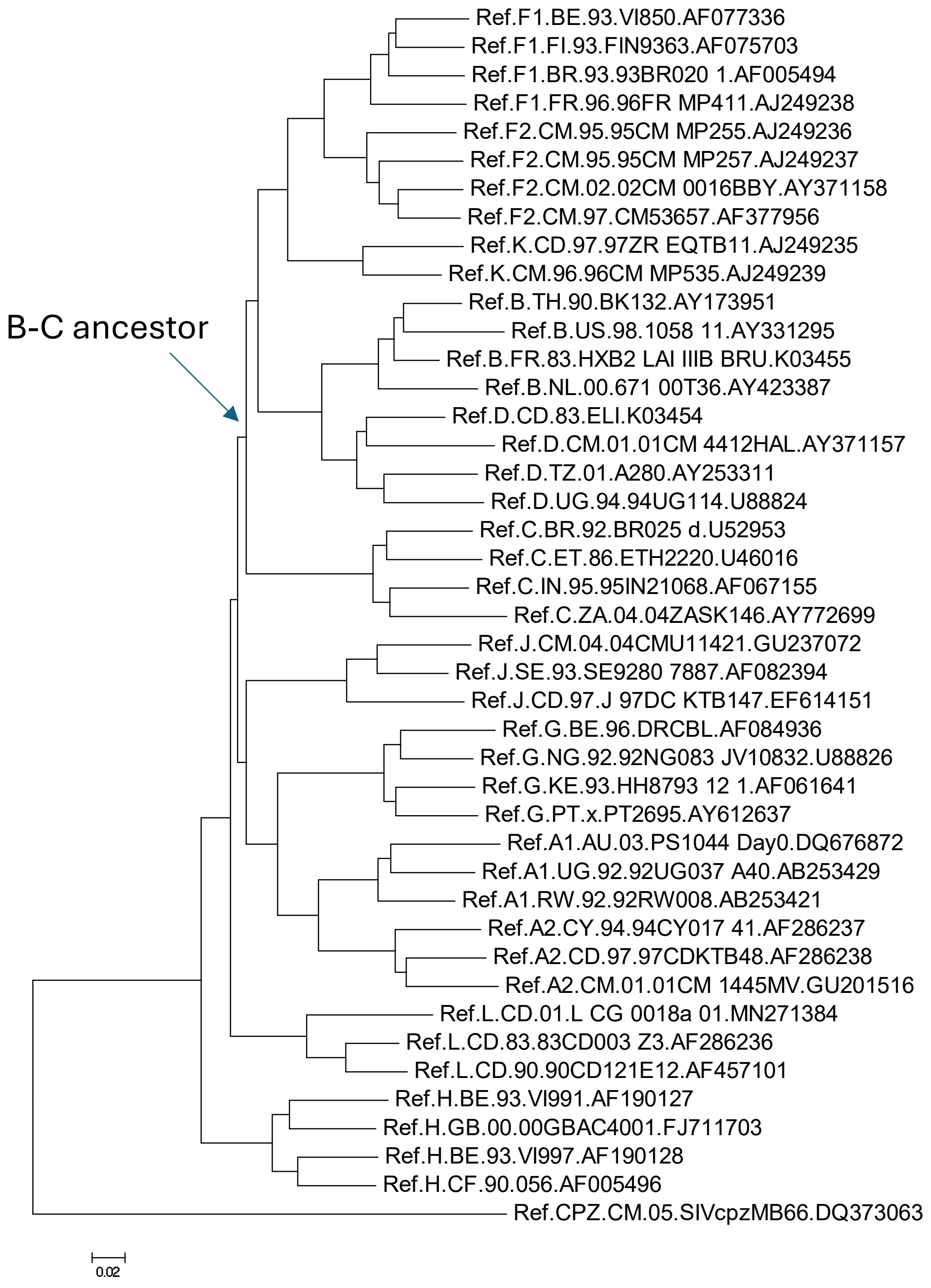

Figure S1. Phylogenetic tree of HIV-1 group M subtype references used to reconstruct the B-C ancestral sequence. The tree is rooted with the chimpanzee (Pan troglodytes troglodytes) SIV MB66 sequence. The tree was constructed with IQ-Tree using the best-fit evolutionary model selected by the program. The node representing the hypothetical most recent common ancestor of viruses of subtypes B and C is signaled with an arrow.

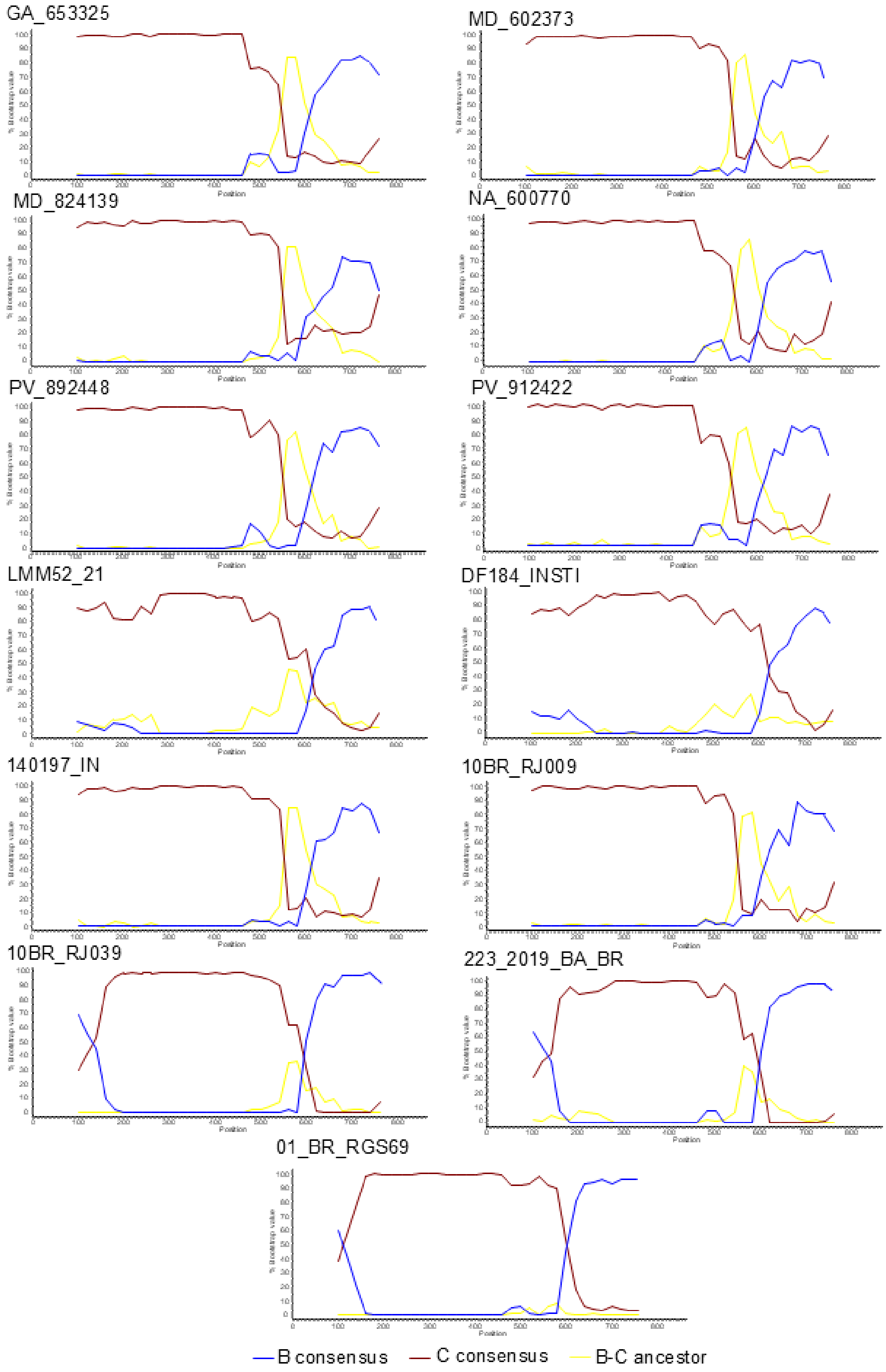

Figure S2. Bootscan analyses of integrase sequences of BC3 cluster and related viruses and of viruses classified as CRF146_BC at the HIV Sequence Database. The horizontal axis represents HXB2 nt positions starting from the 5’ end of integrase and the vertical axis represents the bootstrap values with which the query sequence groups with the reference sequences, which are 75% consensuses of subtype B and the Brazilian subtype C strain, and a reconstructed BC ancestor sequence used as outgroup. Trees were constructed with the neighbor-joining method using Kimura 2-parameter distances. A window of 200 nt was used, moving in 20 nt increments.

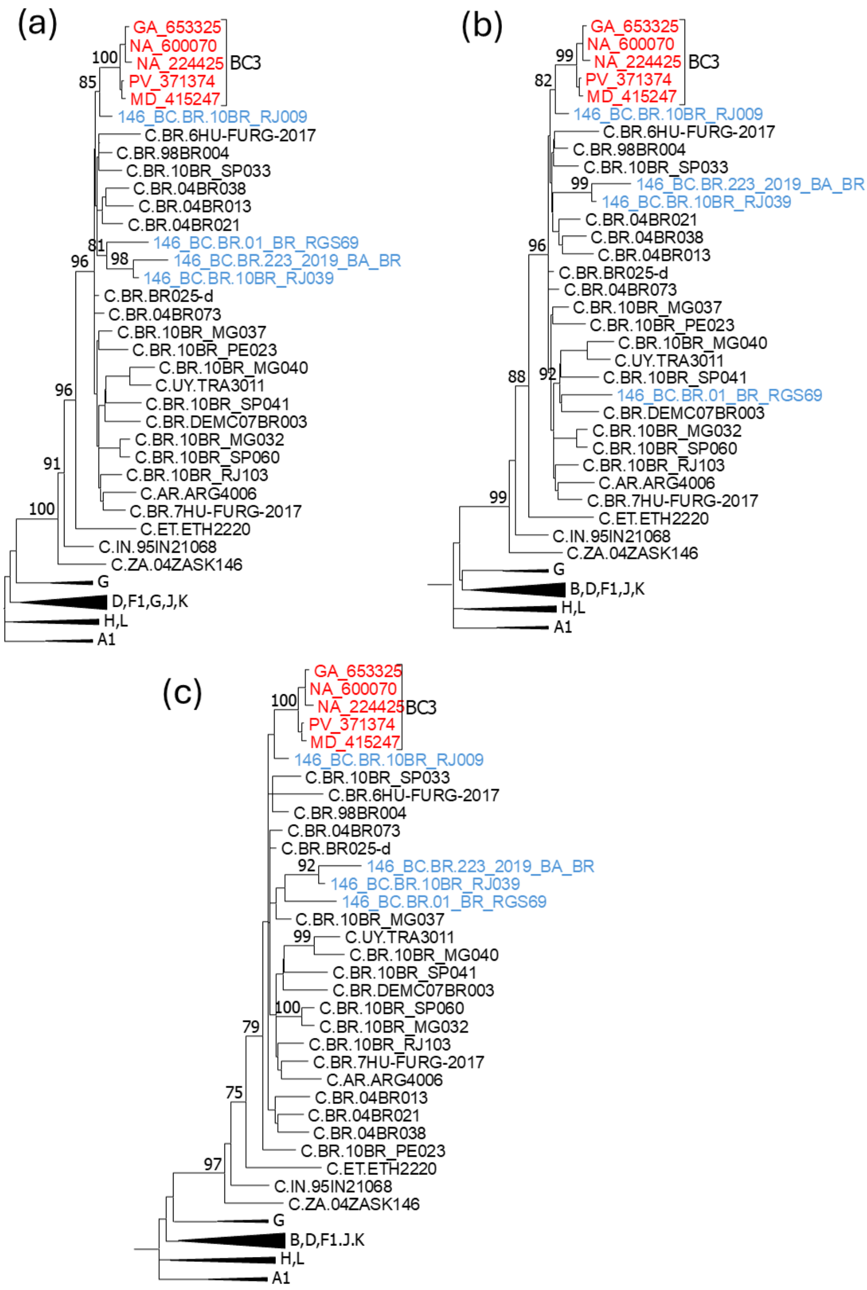

Figure S3. Phylogenetic trees of concatenated subtype C fragments in integrase and surrounding segments of BC3 cluster viruses and viruses previously classified as CRF146_BC. The tree was constructed with IQ-Tree, with assessment of node supports with (a) UFB, (b) aLRT SH-like; and (c) standard bootstrapping procedures. Names of samples of the BC3 cluster are shown in red and those previously classified as CRF146_C are shown in blue. In addition to the viruses of interest, sequences of subtype B references and of all subtype C viruses from South America with NFLG sequences available at the HIV Sequence Database are included. The tree is rooted with a reconstructed BC ancestral sequence. Only node support values ≥70% are shown.

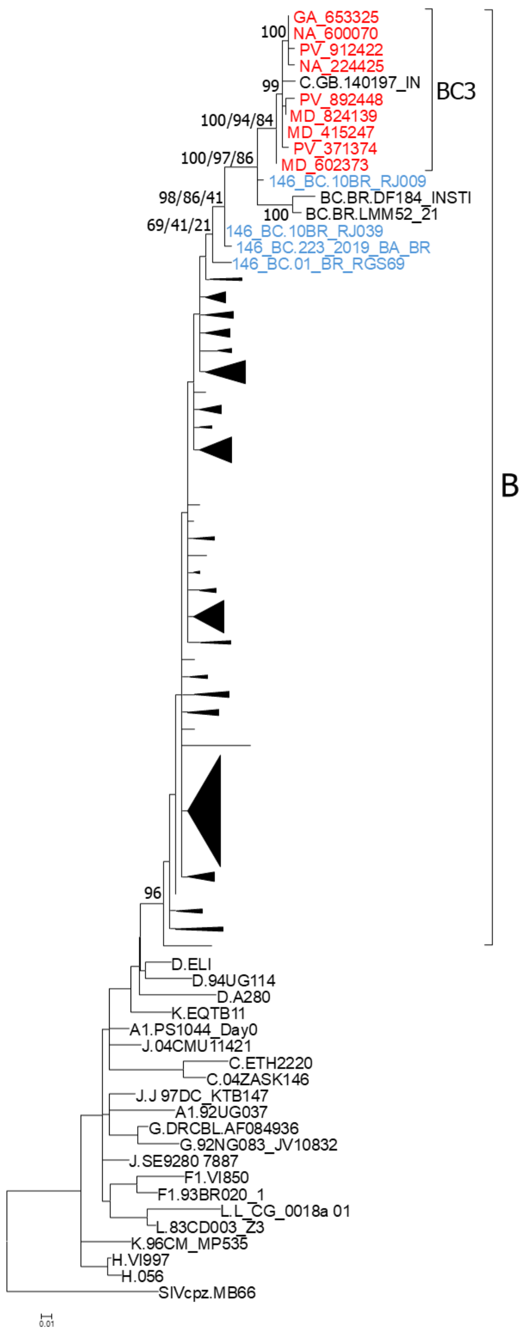

Figure S4. Phylogenetic tree of the subtype B fragment of integrase of BC3 cluster and related viruses and viruses previously classified as CRF146_BC. The tree was constructed with IQ-Tree. Names of viruses of the BC3 cluster are shown in red and those classified as CRF146_BC at the HIV Sequence Database are shown in blue. Subclades within the subtype B clade are compressed. In addition to the viruses of interest, sequences of HIV-1 group M subtype references and of all subtype B viruses from Brazil with NFLG sequences available at the HIV Sequence Database are included. The topology of the shown tree corresponds to that obtained using UFB for node support assessment, which coincides for the viruses of interest with those obtained using aLRT SH-like and standard bootstrapping procedures. Support values for the nodes of interest are shown in this order: UFB/aLRT SH-like/standard bootstrap values.
